# Anticoagulation Strategies during Extracorporeal Membrane Oxygenation: A Narrative Review

**DOI:** 10.3390/jcm11175147

**Published:** 2022-08-31

**Authors:** Sasa Rajsic, Robert Breitkopf, Dragana Jadzic, Marina Popovic Krneta, Helmuth Tauber, Benedikt Treml

**Affiliations:** 1 Department of Anaesthesiology and Intensive Care Medicine, Medical University Innsbruck, 6020 Innsbruck, Austria; 2 Anaesthesia and Intensive Care Department, Pain Therapy Service, Cagliari University, 09042 Cagliari, Italy; 3 Institute for Oncology and Radiology of Serbia, 11000 Belgrade, Serbia

**Keywords:** adverse events, anticoagulation, complications, extracorporeal life support, ECMO, inflammation, monitoring, mortality, future directions

## Abstract

The development of extracorporeal life support technology has added a new dimension to the care of critically ill patients who fail conventional treatment options. Extracorporeal membrane oxygenation (ECMO)—specialized temporary life support for patients with severe cardiac or pulmonary failure—plays a role in bridging the time for organ recovery, transplant, or permanent assistance. The overall patient outcome is dependent on the underlying disease, comorbidities, patient reaction to critical illness, and potential adverse events during ECMO. Moreover, the contact of the blood with the large artificial surface of an extracorporeal system circuit triggers complex inflammatory and coagulation responses. These processes may further lead to endothelial injury and disrupted microcirculation with consequent end-organ dysfunction and the development of adverse events like thromboembolism. Therefore, systemic anticoagulation is considered crucial to alleviate the risk of thrombosis and failure of ECMO circuit components. The gold standard and most used anticoagulant during extracorporeal life support is unfractionated heparin, with all its benefits and disadvantages. However, therapeutic anticoagulation of a critically ill patient carries the risk of clinically relevant bleeding with the potential for permanent injury or death. Similarly, thrombotic events may occur. Therefore, different anticoagulation strategies are employed, while the monitoring and the balance of procoagulant and anticoagulatory factors is of immense importance. This narrative review summarizes the most recent considerations on anticoagulation during ECMO support, with a special focus on anticoagulation monitoring and future directions.

## 1. Introduction

The development of extracorporeal life support modalities has added a new dimension to the care of critically ill patients who fail conventional treatment options. Extracorporeal circuits, like those used in hemodialysis, cardiopulmonary bypass, ventricular assist devices, extracorporeal membrane oxygenation (ECMO), and therapeutic apheresis, are common in modern medicine.

Extracorporeal membrane oxygenation presents specialized temporary life support for patients with severe cardiac or pulmonary failure, bridging time for organ recovery, transplant, or permanent assistance. The beginning of ECMO support dates from 1971, when the first prolonged extracorporeal oxygenation and perfusion were used in the case of a patient with severe acute respiratory distress syndrome (ARDS) [1]. Over the last decade, the indications for ECMO support have expanded beyond severe respiratory failure and refractory cardiogenic shock [2] to include an assortment of clinical presentations, including a bridge to heart or lung transplantation [3], extracorporeal cardiopulmonary reanimation (ECPR) [4], resuscitation of patients with severe traumas [5], and rewarming due to accidental deep hypothermia [6]. Recently, the use of ECMO support for out-of-hospital cardiac arrest has been popularized in some countries, with reported improvements in outcome [7,8,9,10].

The outbreak of coronavirus disease 2019 (COVID-19) led to a significant increase in ECMO use and, based on the Extracorporeal Life Support Organization (ELSO) data, almost 173,000 ECMO runs had been reported by the end of 2021, with 17,777 runs in the last year. The overall in-hospital survival was 54% [11].

The overall survival is dependent on the underlying disease, comorbidities, patient reaction to critical illness, and potential adverse events during ECMO support. The initiation of ECMO support is associated with complex inflammatory and coagulation responses, as a reaction to the blood encountering the large artificial surface of an extracorporeal system circuit [12]. These processes may further lead to endothelial injury and disrupted microcirculation with consequent end-organ dysfunction and the need for systemic anticoagulation [12,13].

Therefore, anticoagulation is considered crucial to reduce the risk of thrombosis and failure of circuit components. Unfractionated heparin (UFH) is the gold standard and most-used anticoagulant during extracorporeal life support. It achieves anticoagulatory effects by enhancing the activity of antithrombin, which results in downregulation of thrombin and activated factor X (factor Xa) [14]. However, therapeutic anticoagulation of a critically ill patient carries the risk of clinically relevant bleeding with the potential for permanent injury or death [15,16,17]. Similarly, thrombotic events may occur [18,19]. Therefore, the monitoring of anticoagulation and the balance of procoagulant and anticoagulatory factors is of immense importance [17].

This review outlines the most recent considerations for anticoagulation and summarizes and discusses various anticoagulation strategies during ECMO support, with a special focus on anticoagulation monitoring. Furthermore, we emphasize the significance of future directions of extracorporeal life support, with a brief overview of costs.

## 2. ECMO Configurations and Circuits

There are two main ECMO configurations: venoarterial (va-ECMO), used for a refractory cardiogenic shock, and venovenous (vv-ECMO), used for a severe respiratory failure, both of which can be subject to several modifications [20]. Venoarterial ECMO combines adequate oxygen delivery and carbon dioxide removal with circulatory support [21]. Vascular access is obtained by the placement of a drainage cannula in a large central vein supplying the ECMO system with patient blood. A second cannula returns the oxygenated blood either to the venous (vv-ECMO) or the arterial system (va-ECMO).

The traditional ECMO circuit utilizes technology as a cardiopulmonary bypass, i.e., it is a closed circuit with a membrane-type gas-exchange system [21]. The main distinctions between those two extracorporeal life modalities are in the duration of support, the existence of a venous reservoir, the air–blood interface, and the cardiotomy reservoir. Cardiopulmonary bypass is usually only employed for the duration of surgery, while ECMO support may be needed for weeks or even months [21].

**Figure 1:**
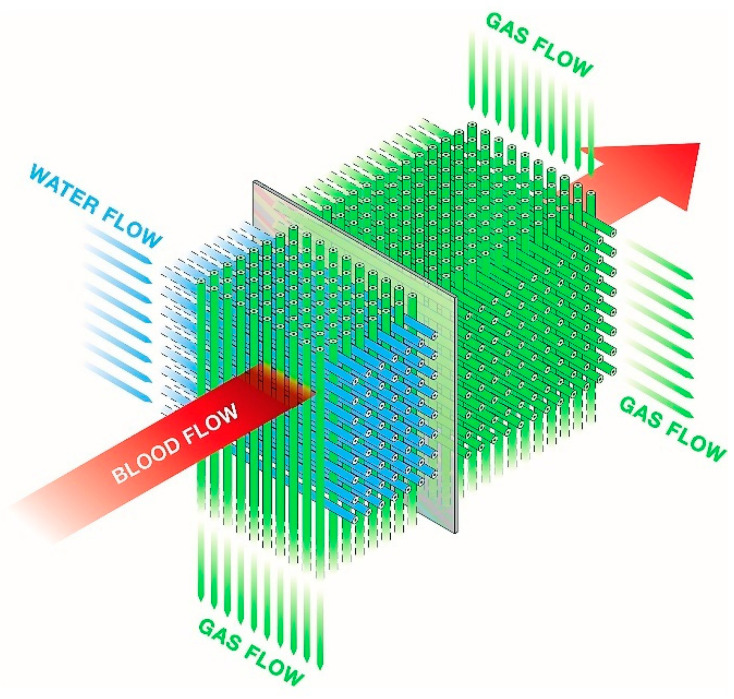
Schematic presentation of a diffusion membrane showing blood flow between the gas and water-filled network of hollow fibers. With permission of Maquet.

The ECMO circuit consists of three main components: the pump, the gas, and the heat-exchange device connected with the polyvinyl chloride tubing (usually UFH coated). The earlier roller blood pumps have now been exchanged with more advanced and magnetically actuated centrifugal pumps that control the required blood flow [22,23]. Gas-exchange devices, fulfilling the patient’s metabolic needs for oxygen and the removal of carbon dioxide, evolved from direct air–blood contact systems to membrane-type gas-exchange devices (oxygenators). Since the early 2000s, polymethylpentene hollow fiber membranes are increasingly used, where the gas is ventilated through hollow fiber bundles and the blood circulates around the fibers (Figure 1) [21,24].

The amount of oxygen in the gas mixture depends on the metabolic need of the patient and can be increased up to 100% oxygen. The gas–blood flow ratio is typically adjusted to maintain normocapnia. Increasing the sweep gas flow will lead to an increased clearance of carbon dioxide while not altering oxygenation. Decreasing the sweep flow will result in increased partial pressure of carbon dioxide in the blood [21].

Keeping the circulating blood pressure in the oxygenator higher than the pressure of the circulating gas is of utmost importance to prevent the passage of air bubbles across the membrane, which can result in air embolism. Therefore, the oxygenator device should always be located below the level of the patient’s heart. Furthermore, insertion or removal of central catheters or interventions with the opening of blood vessels could result in aspiration of air by the ECMO system [21,25].

Finally, the third component of the ECMO system is a heat exchanger preventing circuit-related heat dispersion but also giving the option of a targeted temperature management, for example, in the treatment of sepsis, metabolic crisis, rewarming of accidental hypothermia, or as therapeutic hypothermia after cardiac arrest. The heat exchanger may be integrated into the gas-exchange device or be a separate component. It is usually based on nonpermeable hollow fiber bundles with circulating nonsterile water (Figure 1) [21].

## 3. Inflammation, Coagulation, and ECMO

The normal hemostasis in critically ill patients receiving ECMO support is distorted. Surgical trauma (ECMO implantation or cardiac surgery) and exposure of blood to the large surfaces of the ECMO circuits initiate and propagate immediate inflammatory response and activation of the coagulation cascade. Furthermore, the complexity of critical illness and its inflammatory response may additionally imbalance patient hemostasis [12]. The systemic inflammatory response in patients receiving cardiopulmonary bypass is well established and discussed extensively in the literature [26,27,28,29,30], but the information on this complex and multifaceted inflammatory response to ECMO support is still limited.

Several humoral and cellular systems are involved in complex interactions between inflammation and coagulation during ECMO support. Acute inflammation initiates clotting, compromises the fibrinolytic system, and reduces the activity of natural anticoagulant mechanisms. Moreover, endotoxin, IL-1β, and tumor necrosis factor-α (TNF-α) downregulate thrombomodulin and neutrophil elastase cleaves thrombomodulin from the endothelial cell surfaces [31,32]. P-selectin and E-selectin are synthesized or expressed on endothelial and platelet surfaces. Tissue factor, from the cell surface of leucocytes and monocytes, is induced by endotoxin, CD40 ligand, or TNF-α. It further binds factor VIIa and converts factor X to its activated form (Xa), which together with factor Va generates thrombin from prothrombin [33]. Additionally, inflammation reduces protein C levels, probably due to a combination of consumption and associated liver dysfunction with a consequent nonactivation of factor Va leading to the stabilization of prothrombin activation complexes [34]. Increased C-reactive protein levels facilitate monocyte–endothelial cell interactions, promote plasminogen activator inhibitor-1 and tissue factor formation, and induce complement activation [35,36,37]. The activation of platelets, key elements of hemostasis and inflammation, occurs as a result of complement activation and thrombin generation with a consequent release of a variety of mediators (proinflammatory cytokines, chemokines, adhesion factors, proteases, hemostatic factors, etc.). This all together plays a role in the development of a systemic inflammatory response [38,39,40,41].

Antithrombin is inactivated and/or consumed, while the levels of vascular heparin-like molecules may be reduced due to neutrophil activation products and inflammatory cytokines [42,43,44]. Finally, detailed information on the role of the fibrinolytic system in patients receiving ECMO support is still lacking, but recent studies reported on the association between increased fibrinolysis and bleeding complications [45,46].

Furthermore, different configurations of ECMO may also alter hemostasis. In the prospective HECTIC trial, the rate of thrombosis in va-ECMO was around 40%, with rates twice as high in vv-ECMO [47]. Moreover, in an ex vivo model, ECMO flow rates below 1.5 L/min were shown to decrease platelet aggregation, weaken clot firmness, and surprisingly increase hemolysis (despite the lower pump speed) [48]. Given the sparsity of evidence for low ECMO flows in humans, we establish more anticoagulation at lower flow rates (e.g., ACT 150–170 s at 2–3 L/min). Moreover, we use the same anticoagulation protocol per se in va- and vv-ECMO configurations but strive to tailor anticoagulation to each patient using viscoelastic monitoring on a routine basis.

**Figure 2:**
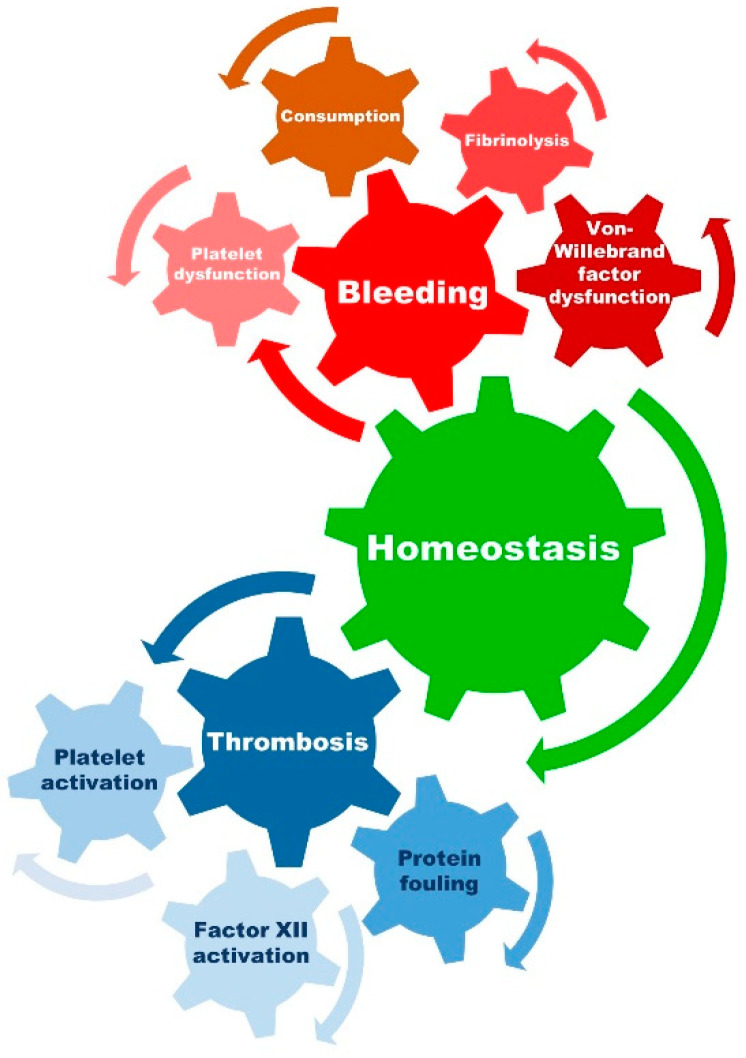
Presentation of prothrombotic and prohemorrhagic factors with an influence on homeostasis. Achieving a balance between the risk of bleeding and thrombosis is both critical and complex in patients receiving ECMO support. Aside from the initiation and propagation of the inflammatory response (proinflammatory state) and the activation of the coagulation cascade (prothrombotic state), ECMO may also lead to platelet dysfunction, fibrinolysis, malfunction of von Willebrand factor, and consumption of coagulation factors leading to a prohemorrhagic state.

Therefore, homeostasis and the balance between the procoagulant and anticoagulatory factors are crucial to avoid hemorrhagic or thromboembolic complications, and for the patency of the extracorporeal circuit and its components (see Figure 2).

Interestingly, within the first 10 min after ECMO support initiation and contact of blood with the artificial surfaces, factor XII cleaves into factor XIIa and XIIf. Factor XIIa has an important role in the activation of kallikrein and bradykinin, both strong drivers of inflammation and coagulation [49,50,51]. The role of bradykinin in inflammation may be even more interesting in va-ECMO with the lungs, the major site of bradykinin inactivation, being bypassed. As a response to the role of factor XII, its neutralization may lead to a reduction in inflammation, which has been recently shown in ex vivo and animal ECMO models with the use of a plasma protease factor XII function-neutralizing antibodies [52,53]. Further studies focused on potential uses in humans are warranted.

Lastly, it should be mentioned that, like any exposure to mechanical support devices, patients on ECMO support can develop increased human leukocyte antigen (HLA) sensitization, which is of relevance in bridge-to-transplant therapeutic considerations [54].

Given the above, systemic anticoagulation and coagulation monitoring are of immense importance for adverse events prevention in patients receiving ECMO support. The association of inflammation and thrombosis, known as thromboinflammation, is well reported in the literature, especially in COVID-19 patients [55,56]. Hyperinflammation may lead to a limitation of the UFH effect by decreasing antithrombin levels or increasing heparin binding to acute phase proteins [42,43,44]. A recent report on a possible association between bleeding and unintended excessive anticoagulation in ECMO patients without hyperinflammation remains to be confirmed in larger cohorts [13]. Finally, despite the extensive development of anticoagulants, ECMO pumps, oxygenators, and tubing systems, the systemic inflammatory response syndrome and distorted hemostasis remain a clinical concern. It is still unclear if the extent of inflammation may also benefit the patient, beyond its deleterious effects. To warrant a more detailed understanding of the underlying pathophysiological processes, the reporting on inflammatory response during ECMO support should be improved in forthcoming studies.

## 4. Anticoagulation Strategies

Extracorporeal life support continues to be the last resort for critically ill patients with cardiopulmonary failure. Despite remarkable developments in technology, there is no satisfactory circuit design that can eliminate the need for systemic anticoagulation. The state-of-the-art tubing systems contain modified surfaces, with the aim of overcoming the blood-circuit surface interaction by mimicking the endothelium and having antithrombotic properties [57]. However, these systems are yet to eliminate the risk of inflammation and coagulation cascade activation. Thus, systemic anticoagulation is still necessary to reduce the risk of thrombosis and maintain the patency of the extracorporeal circuit and its components [21].

There is ongoing research and limited evidence to guide the optimal anticoagulant selection, dosing strategy, and monitoring in the ECMO setting [58]. Furthermore, anticoagulant-free ECMO support is being discussed, and Olson et al. systematized the evidence, concluding that the incidence of thrombosis was comparable to patients receiving systemic anticoagulation [59]. In the following paragraphs, an overview of the most recent evidence on anticoagulation during ECMO support is presented with a summary of the commonly used agents in Table 1.

### 4.1. Heparin Products

**Figure 3:**
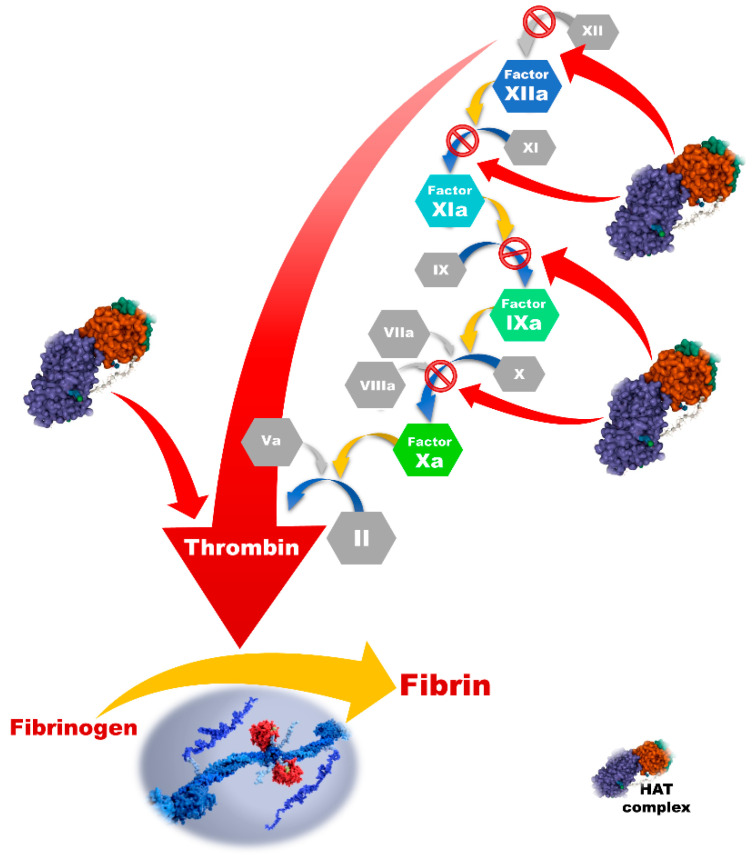
The heparin–antithrombin–thrombin (HAT) complex inactivates the coagulation factors, leading to the blockade of the fibrinogen conversion to fibrin. The red arrows starting from the HAT complexes show the place of its action on factors XIIa, XIa, IXa, Xa, and IIa. At the bottom of the figure, the fibrinogen molecule (blue) is shown with two thrombin molecules (red) catalyzing its transition to the active form, fibrin. (Adapted with permission from Dreamstime.com. 2022, Illustration 183970741 ©Juan Gaertner and Illustration 233379397 ©Volodymyr Dvornyk, accessed on 10 August 2022).

Heparin, a mixture of heterogeneous glycosaminoglycans, was isolated from the dog liver in 1916 (“*hepar*” is Greek for liver) [60,61]. Nowadays, it is predominantly acquired from porcine intestinal mucosa or bovine lung and requires an extensive process of purification during pharmaceutical preparation [62,63]. Heparin’s major antithrombotic effect is based on a complex formation with antithrombin, which is further responsible for the inactivation of thrombin, activated factor X, and other coagulation factors [64]. Approximately one-third of an administered commercial heparin binds to the antithrombin, forming the active fraction, which is accountable for its anticoagulant effects [65,66]. Heparin does not have any impact on thrombin formation or inhibition of the thrombin–fibrin complex. It inactivates thrombin after it is already formed [14]. In this way, the conversion of fibrinogen to fibrin is blocked, and the formation of clots is prevented with the prolonged clotting time of blood (Figure 3) [67].

Based on its molecular weight (ranging from 3000 to 30,000 Da), heparin can be divided into UFH, containing all the fractions of molecules independent of molecular weight, and low-molecular-weight heparin (LMWH), with an average molecular weight of less than 8000 Da [63,68]. The main difference is seen in their pharmacokinetics, as the UFH needs to be monitored and the dosages adjusted, while LMWH preparations may be used without laboratory monitoring in selected patient groups [69]. Unfractionated heparin has an immediate onset of action when administered intravenously, and is metabolized by the reticuloendothelial system and the kidneys [67].

#### 4.1.1. Unfractionated Heparin (UFH)

Due to its rapid onset and possible immediate reversal, UFH is the most frequently used drug for anticoagulation of patients undergoing ECMO support globally [70]. The ELSO anticoagulation guidelines recommend an initial bolus at the time of cannulation (50–100 IU/kg), and continuous intravenous infusion during the whole ECMO course (initiated with 5–20 IU/kg/h, and usually achieving the therapeutic anticoagulation goal at 20–50 IU/kg/h). If the patient underwent transthoracic cannulation, a cardiopulmonary bypass procedure, or in cases of severe coagulopathy and active bleeding, the UFH bolus can be adapted and the continuous application of systemic anticoagulation may be delayed [58]. After ECMO is initiated, anticoagulation monitoring should be started and the rate of UFH infusion adapted accordingly. The most commonly used point-of-care and laboratory tests include the activated partial thromboplastin time (aPTT), activated clotting time (ACT), antifactor Xa activity levels (anti-Xa), blood concentration of drugs, and viscoelastic tests. The most commonly used test for anticoagulation monitoring is the ACT measurement [21]. The recommended anticoagulation goal ranges between 180 and 220 s, varying between centers and based on the patients’ clinical characteristics [71,72]. Moreover, the use of continuous renal replacement therapy, increased urine output, administration of platelets, or the presence of hyperinflammation may further limit the anticoagulation effect of heparin and result in the need for increased UFH dosage, according to goal-directed anticoagulation [58,73]. Additional limitations of heparin use include heparin resistance and heparin-induced thrombocytopenia (HIT), which are elucidated in later paragraphs.

#### 4.1.2. Low-Molecular-Weight Heparins (LMWH)

Low-molecular-weight heparins (LMWH, e.g., enoxaparin, dalteparin, tinzaparin, nadroparin) are derived from heparin as a result of depolymerization generating fragments that are nearly a third of the size of a heparin molecule [68]. These fragments predominantly act on factor Xa, in contrast to the UFH (thrombin). However, the main anticoagulant effect, i.e., activating antithrombin, is the same as that of UFH. Monitoring of LMWH therapy is usually performed by measuring the concentration of anti-Xa levels [69]. Finally, due to its more predictable pharmacokinetic profile and ease of administration, this anticoagulant is widely used in the prevention and treatment of venous thromboembolism [74].

As a relevant part of ECMO, patients suffer from hemorrhagic diathesis, and bleeding is still a major concern. The risk of life-threatening hemorrhage, blood product transfusion, and the risk of thromboembolic events should be taken into consideration when initiating systemic anticoagulation. In well-selected patients, the use of reduced anticoagulation by using low-dose heparin only or different dosages of LMWH is being investigated [75,76,77,78].

Krueger et al. reported on vv-ECMO patients with respiratory failure who received only prophylactic subcutaneous anticoagulation (enoxaparin, 1 × 40 mg/day), the same as all other critical care patients in this department [75]. The cannulas were not coated in most patients. Within of total 560 ECMO support days and a median duration of 7 days, 34% of patients died during ECMO support, 6.5% experienced severe thrombotic or thromboembolic events, and in three cases, the centrifugal pump stopped due to thrombotic occlusion, with consequent emergency pump exchange. No changes in the oxygenator were needed, and bleeding occurred in 30% of patients. Based on the findings from 61 patients, the authors conclude that vv-ECMO with only prophylactic anticoagulation may be feasible [75]. Another study compared the use of UFH and enoxaparin in a dose regime of 2 × 0.5 mg/kg/day, without guidance by anti-Xa levels [77]. Based on the mixed vv- and va-ECMO sample of 102 lung transplant patients, the authors concluded that there is no difference in risk of bleeding when comparing the use of LMWH and UFH for systemic anticoagulation. Moreover, patients receiving LMWH had a lower risk of thromboembolic events [77]. Finally, Wiegele et al. compared the use of heparin and enoxaparin (2 × 4000 IU, anti-Xa level monitored) in COVID-19 patients and concluded that the subcutaneously administered enoxaparin is superior (given the rate of adverse events) when compared to UFH, and may be seen as a possible anticoagulation strategy in COVID-19 patients requiring ECMO support [78].

The main limitations of the above studies are the rather small study samples and their single-center and retrospective design, respectively. Moreover, without routine screening for thromboembolic events, the real incidence may be underestimated [79].

However, these findings imply the urgent need for further prospective randomized trials to shed light on the efficacy and safety of LMWH-based anticoagulation strategies in patients receiving ECMO support in order to provide definite conclusions and recommendations for the future.

#### 4.1.3. Heparin-Coated ECMO Circuits

Heparin-coated ECMO circuits became commercially available in 1983, aiming to reduce the risk of bleeding by lowering the need for systemic anticoagulation [80]. The mechanism of action is based on the covalent binding of heparin to the artificial surfaces of ECMO circuits and imitation of the antithrombogenic effects of heparan sulfate at the endothelium. The coated surface can be considered stable, releasing only insignificant amounts of heparin into the circulating blood. Moreover, it is highly thromboresistant with the capacity to prevent clotting of nonanticoagulated blood [80]. The benefits of heparin-coated circuits are reduced humoral and cellular activation, lessened complement system activation, and lower production of oxygen radicals. Furthermore, a reduced activation of neutrophils and platelets lowers the consequent inflammatory response, which in combination with a lower rate of pulmonary and central nervous system complications, may shorten the hospital stay [81]. However, as these circuits contain heparin, they may not be employed in patients with suspected or confirmed HIT, and in these patients, an already existing system should be exchanged for a heparin-free one [82].

#### 4.1.4. Heparin Resistance and Antithrombin Deficiency

Heparin resistance is a specific clinical concern in patients on ECMO support. It may be defined as a failure to achieve a specified anticoagulation level despite the use of increasing heparin doses, as identified by anticoagulation monitoring (ACT, aPTT, antifactor Xa) or thrombosis occurrence [44]. The threshold dose for heparin resistance in ECMO patients is still not well defined. The missing consensus on the appropriate anticoagulation target level and the best methods to measure heparin effects further complicate the interpretation. An arbitrary threshold of 35,000 U of heparin per day in ECMO patients or more than 500 U/kg in cases of cardiopulmonary bypass is used in some studies [44]. However, such a threshold does not take into account factors possibly influencing a heparin efficacy, e.g., the body mass index, sex, prothrombotic states (i.e., thromboinflammation as in COVID-19, sepsis, etc.), or antithrombin deficiency.

The fluctuation of heparin response among patients has a pharmacokinetic and biochemical basis. Firstly, the heparin–antithrombin complex is not able to inactivate factor Xa bound to platelets and bounded factor Va, in addition to the well-known incompetence to inactivate the fibrin-bound thrombin. Secondly, heparin may be bound to plasma proteins, mostly to those of acute-phase reactants, with a consequently reduced biologic availability, increased heparin clearance, drug interactions, and congenital or acquired antithrombin deficiency. This becomes especially important in critically ill patients, with hyperinflammation or sepsis [83].

Antithrombin deficiency is a commonly reported cause of heparin resistance, due to heparin’s primary mechanism of anticoagulation. Acquired antithrombin deficiency is common in a variety of clinical presentations including liver disease, sepsis, nephrotic syndrome, malnutrition, increased consumption during bleeding or disseminated intravascular coagulation, employment of extracorporeal systems, and the use of heparin. The ECMO-related antithrombin deficiency is frequently seen upon ECMO initiation and may be attributed to a combination of its reduced synthesis and accelerated consumption [44].

Recent studies on heparin resistance management strategies recommended anti-Xa measurements, especially in patients with substantial inflammation, as the use of aPTT may be biased. If the measured anti-Xa levels are low, the UFH dose should be increased to achieve the goal anti-Xa level (0.3–0.7 IU/mL). Some centers substitute antithrombin, which is still a subject of ongoing debate. In case of persisting heparin resistance, other anticoagulants can be successfully employed (direct thrombin inhibitors, direct thrombin inhibitors) [17,44]. However, the minimal antithrombin activity required for sufficient heparin function is still unknown and clear recommendations based on strong evidence are missing. Further research on routine antithrombin monitoring and substitution is assured.

#### 4.1.5. Heparin-Induced Thrombocytopenia

Heparin-induced thrombocytopenia type II is a severe and potentially life-threatening immune adverse reaction characterized by thrombocytopenia and thrombosis. Most commonly, it appears within the first 10 days of heparin therapy, with an associated platelet count fall (more than 50%) and hypercoagulability. It is the result of antibody formation (IgG) against heparin and platelet factor 4 (PF4) complex [84]. The emergent heparin–PF4–IgG complex activates platelets leading to the release of prothrombotic platelet-derived mediators, consumption of platelets, and finally, thrombocytopenia [84]. The prevalence of HIT varies from 0.1% to 5% in patients receiving heparin, with approximately 25% to 50% of patients developing complications [85]. The reported incidence in patients on ECMO ranges from 0.36% and 8.3% [86].

Both clinical and serological features are used for the diagnosis of HIT. Aside from the few developed clinical scoring systems (e.g., 4T-score), clinical manifestations include platelet count fall (>50%), onset between 5 and 10 days, new thrombosis, and exclusion of other possible causes of thrombocytopenia [85]. Serological investigation and antibody detection is necessary in case of high suspicion of HIT. Immunoassays can detect anti-PF4-heparin antibodies, with enzyme-linked immunosorbent assays (ELISA) still being the gold standard. Functional assays or platelet activation assays can further investigate if these antibodies are able to activate platelets in the presence of heparin (serotonin release assay (SRA); heparin-induced platelet activation (HIPA)) having the highest sensitivity in diagnosis of a HIT [85].

However, diagnosing HIT in patients on ECMO support is challenging and requires clinician awareness since the blood response to ECMO can mimic HIT (e.g., thrombocytopenia, thrombosis, sepsis, disseminated intravascular coagulation, etc.). In case of suspected HIT, heparin therapy should be immediately suspended, and alternative anticoagulation started. It is important to mention that the heparin-coated ECMO components need to be replaced as well. Moreover, we recommend 24/7 availability of heparin-free ECMO circuits for prompt replacement. The alternative anticoagulation is strongly recommended and it may include DTI and factor Xa inhibitors [86]. Failure to pursue the anticoagulation of a patient with high suspicion of HIT may lead to a clinically significant thrombotic event as these patients have a 30-fold increased risk of thrombosis compared to the normal population [86].

### 4.2. Direct Thrombin Inhibitors (DTI)

Direct thrombin inhibitors present an alternative anticoagulation strategy, which is still predominantly reserved for patients with suspected or confirmed HIT and heparin-resistance, or the development of thrombosis while on UFH therapy [87]. This relatively new class of drugs has various advantages over UFH, and the two most frequently used drugs are argatroban and bivalirudin. These anticoagulants are directly bonded to the active site of thrombin, both free circulating, and (unlike heparin) also fibrin bounded. They bind to other plasma proteins to a lesser extent, making pharmacokinetics more predictable and the process antithrombin independent [88].

Argatroban is characterized by fast liver-dependent metabolization and a half-life time of approximately 45 min, which is of additional value in ECMO patients with a high risk of bleeding and lack of a specific antidote [89]. Continuous intravenous infusions are usually maintained with 0.1–0.7 mcg/kg/min and further adjusted based on the anticoagulation monitoring [21]. However, our opinion is that argatroban dosing should be performed with caution, especially in patients with hepatic impairment. These patients may need a maintenance does as low as 0.1–0.2 mcg/kg/min. Fisser et al. found that argatroban is noninferior to UFH, with respect to thrombotic and bleeding events, concluding that it can be safely used in patients receiving vv-ECMO support. However, the direct drug costs of argatroban were higher [90]. A recent systematic review with 307 patients reported comparable rates of bleeding and thromboembolic complications in patients treated with argatroban and UFH, its safety and efficacy being limited by the scarcity of studies [91]. Finally, the results of an ongoing prospective randomized controlled trial on the safety and feasibility of argatroban in patients with ECMO support are expected at the end of 2024 (NCT05226442).

The second representative of the DTI group is bivalirudin, with a rather short half-life of 25 min. It undergoes dual elimination, via proteolytic degeneration and partial renal excretion, completely independent of the liver [92,93,94]. Reported initial doses in adult patients range from 0.03 mg/kg/h [92] to 0.5 mg/kg/h [95], with an average of 0.27 ± 0.37 mg/kg/h [96], adjusted according to the anticoagulation monitoring. Ranucci et al. found bivalirudin to be safe in postcardiotomy ECMO patients. Moreover, it may have a better coagulation profile, with fewer hemorrhagic events and transfusions of blood products with a comparable thromboembolic complications rate [92]. A recent systematic review and meta-analysis based on 10 articles and 847 patients investigated the efficacy and safety of bivalirudin compared to UFH, revealing that bivalirudin may significantly reduce the incidence of major bleeding (in children) and thrombotic events, in-circuit thrombosis, and in-hospital mortality. The authors concluded that bivalirudin can be a safe and feasible alternative to UFH, especially in the case of HIT and heparin resistance [97].

The use of other DTI (dabigatran, desirudin, and lepirudin) is limited by their potential for severe adverse events and less favorable pharmacokinetic profiles compared to the newer DTIs. Therefore, they play no role in anticoagulation during ECMO [98].

Anticoagulation monitoring is usually accomplished using aPTT, ACT, plasma drug concentration, anti-IIa assays, or viscoelastic methods [99]. The main disadvantages of these relatively new drugs include direct drug costs, the lack of a specific reversal agent or antidote, and the potential of destabilization of already existing clots. However, due to the short half-life time, the major disadvantages may have less importance.

Finally, the available information on DTI efficacy originates from rather small studies, and prospective randomized controlled trials with a larger number of patients are still missing. Further research in this area is guaranteed.

### 4.3. Direct and Indirect Factor Xa Inhibitors

Direct factor Xa inhibitors (rivaroxaban, edoxaban, and apixaban) present a new class of anticoagulant drugs with direct inhibition of factor Xa, independent of antithrombin [100]. The use of rivaroxaban is described in one ECMO-related case report, where the main indication was HIT and no other alternatives for anticoagulation were available. The administration was performed via nasogastric tube, 2 × 15 mg/day with anti-Xa monitoring. There were no adverse events recorded, and the patient had a favorable outcome [101]. Finally, there is no information on the use of other direct factor Xa inhibitors during ECMO support. The enteral administration and the paucity of studies limits their use in ECMO.

Fondaparinux, an indirect factor Xa inhibitor with a similar molecular structure to LMWH and UFH, may be also considered as an alternative anticoagulant in case of HIT [86]. Parlar et al. described its successful use (1 × 2.5 mg/day, subcutaneous) in an adult ECMO patient with a high suspicion of HIT. The authors concluded that the use of fondaparinux may be considered an effective and safe alternative treatment in HIT [102].

### 4.4. Heparinoids

Danaparoid is a main representative of the heparinoids, with a well-established antithrombotic activity through antithrombin-mediated factor Xa inhibition and to a lesser extent direct thrombin inactivation [103]. It has minimal effects on the fibrinolytic system and a low tendency to cause hemorrhage [103]. Its use during ECMO support is described in a case report of a patient with severe respiratory failure after massive pulmonary embolism and high clinical HIT suspicion [104]. The patient initially received 400 IU/h for 4 h, then 300 IU/h (0.5–0.8 U/mL anti-Xa factor activity goal) and had a successful outcome.

### 4.5. Factor XIIa Inhibitors, Nitric Oxide, and Circuit Releasing Compounds

The potential novel anticoagulant strategies include the application of factor XIIa inhibitors, nitric oxide, prostacyclin, and other circuit-releasing compounds [58]. Most of these new modalities will still need a certain time to translate from animal to human studies, and more detailed information on future developments may be found in the Future Perspectives section and the conclusions.

### 4.6. Citrate

Critically ill patients frequently develop multiple organ dysfunction, often including acute kidney failure with a need for continuous renal replacement therapy. As anticoagulation is essential to initiate any kind of extracorporeal support, continuous renal replacement therapy commonly employs regional citrate-based anticoagulation. The main mechanism of citrate action is the prevention of the activation of platelets and coagulation cascades by chelation of ionized calcium [105].

As the majority of ECMO patients receive systemic anticoagulation, citrate may be omitted. However, the continuous renal replacement therapy circuits have a lower blood flow rate and heparin coating is usually not employed, increasing the risk of thrombotic events. Therefore, systemic anticoagulation can be supported with the regional citrate anticoagulation, which presents a feasible, safe, and effective technique [105]. In the case of patients with a high risk of bleeding or severe coagulopathy who are not receiving any systemic anticoagulation, continuous renal replacement therapy may be safely used with the regional citrate anticoagulation [106].

The use of citrate for regional ECMO anticoagulation is limited with the citrate clearance, which restricts its use to blood flows that are significantly below the required ECMO blood flow for an adult patient. However, this may have an application in high-risk infants under the age of one, which is being investigated in an ongoing clinical trial (NCT00968565). A complete review of this complex topic is beyond the scope of this work and can be found elsewhere [21].

### 4.7. Antiaggregant Therapy during ECMO Support

A pivotal question for clinicians is the resumption of an indicated dual antiplatelet therapy (DAPT) after recent coronary stent implantation in va-ECMO. Recently, a German group found a similar bleeding rate and mortality with or without DAPT in 93 va-ECMO runs [107]. However, the authors may have missed a small increase in the bleeding rate with DAPT given the high rate of bleeding (60%) and rather short ECMO duration (<3 days). Moreover, patients with DAPT needed more fresh frozen plasma. Such a high bleeding rate may be attributable to an ECMO-induced thrombopenia and thrombopathy. From our clinical experience, low-dose acetylsalicylic acid, unfractionated heparin, or the effect of ECMO-induced thrombopenia and thrombopathy is sufficient for patency of recent implanted coronary stents. However, we recommend completion of DAPT as soon as possible after the removal of ECMO.

### 4.8. Anticoagulation Free ECMO Support

The cessation of systemic anticoagulation in the ECMO setting may reduce the risk of bleeding in patients with severe coagulopathy or a high risk of bleeding. A recent systematic review of studies reporting on patients receiving ECMO support without continuous systemic anticoagulation included 201 patients, mostly treated for acute respiratory distress syndrome or severe cardiogenic shock [59]. During the anticoagulant-free ECMO support, the incidence of ECMO circuit and patient thrombosis was comparable to patients receiving systemic anticoagulation. Regarding bleeding events, due to inconsistency in reporting, no conclusion could be drawn. The consideration of systemic anticoagulation cessation or reduction may be particularly relevant in patients with hemorrhagic diathesis or severe traumas [59]. However, these conclusions are limited by the nature and quality of the included studies (small and nonrandomized studies). Heterogeneity in outcome reporting, without unique definitions and limited adherence to the ELSO definitions of bleeding and thrombosis, makes drawing conclusions difficult [58].

Therefore, in the absence of prospective clinical data and randomized studies, it is still too early to advocate the routine omission of systemic anticoagulation during ECMO support and further research on low systemic anticoagulation is essential.

**Table 1:** Overview of different anticoagulation agents in extracorporeal membrane oxygenation.

Anticoagulant	Mechanism of Action	Monitoring *	Characteristics
Heparin products			
Unfractionated heparin (UFH)	Predominantly inactivating thrombin	aPTT ACT anti-Xa	Half-life: 60–90 min Metabolism: Reticuloendothelial system and the kidneys Antidote: Protamine Advantages: low costs; does not destabilize already existing clots; point-of-care testing possible Disadvantages: Antithrombin dependent; binds to other plasma proteins; heparin resistance and heparin-induced thrombocytopenia
Low-molecular-weight heparins (LMWH)	Predominantly inactivating factor Xa	Anti-Xa	Half-life: 3–6 h Metabolism/elimination: Kidneys Antidote: Protamine (only partially effective) Advantages: low costs; does not destabilize already existing clots; binds less to other plasma proteins; no need for monitoring in selected patient groups Disadvantages: Antithrombin dependent; kidney dysfunction
Direct thrombin inhibitors (DTI)			
Argatroban	Direct thrombin inhibitor	aPTT ACT Blood drug levels	Half-life: 45 min Metabolism: Liver-dependent Antidote: None Advantages: Independent of antithrombin Disadvantages: Potential destabilization of already existing clots; liver dysfunction; higher costs
Bivalirudin	Direct thrombin inhibitor	aPTT ACT	Half-life: 25 min Metabolism: proteolytic degeneration and partial renal excretion Antidote: None Advantages: Independent of antithrombin Disadvantages: Potential destabilization of already existing clots; kidney dysfunction; higher costs
Direct factor Xa inhibitors (Rivaroxaban, edoxaban, apixaban)	Inhibition of factor Xa	anti-Xa	Half-life: 5–12 h Metabolism: Oxidative degradation and hydrolysis Antidote: Andexanet alfa Advantages: Independent of antithrombin Disadvantages: Only case reports available for ECMO patients; formulation for oral application available
Indirect factor Xa inhibitor (Fondaparinux)	Indirect inhibition of factor Xa	anti-Xa	Half-life: 13–21 h Metabolism: Kidney Antidote: None Advantages: Safe in HIT Disadvantages: Antithrombin dependent; only case reports available for ECMO patients
Heparinoids (Danaparoid)	Factor Xa and IIa inhibition	anti-Xa	Half-life: 25 h Metabolism: Kidney Antidote: None Advantages: Safe in HIT Disadvantages: Antithrombin dependent; only case reports available for ECMO patients

* Most commonly applied method. Adapted from: [14,21,64,68,69,88,89,108,109,110].

## 5. Anticoagulation Monitoring

The management and evaluation of coagulation in critically ill patients is one of the greatest challenges, especially when extracorporeal life support and systemic therapeutic anticoagulation are employed. Balancing anticoagulation to prevent hemorrhagic complications against thromboembolic events in already complex and severely ill patients is a subject of ongoing debate. From an international survey of 121 ELSO centers (the vast majority from the USA), 97% of centers employ the serial measurement of ACT and aPTT (94%) for anticoagulation monitoring, 82% perform antithrombin tests, 65% anti-Xa testing, and 43% used viscoelastic methods additional to common assays [70]. Moreover, it is common for different methods to be discordant due to the rather poor correlation between assays, and no single laboratory test has yet been developed that has ideal characteristics for anticoagulation monitoring. The optimal method to measure anticoagulant efficacy during ECMO support is still unknown and, in the following paragraphs, we discuss the monitoring of coagulation and the optimization of patient management during ECMO support.

### 5.1. Activated Clotting Time (ACT)

The ACT remains the primary method for point-of-care heparinization monitoring during extracorporeal life support, cardiac surgery, cardiac catheterization laboratory, dialysis, and vascular surgery, as the prothrombin time and aPTT are immeasurable in presence of high heparin concentrations [21]. This method measures the time needed for a sample of whole blood to clot, by registering the mobility of a magnet during clot formation or the velocity change of magnet movement through clotting blood. ACT is usually repeatedly measured to guide heparin dosing, with a goal of 180–220 s, depending on the bleeding risk [58,111]. The main disadvantages are the poor correlation between heparin blood concentration and anti-Xa measures of heparin activity [112]. Moreover, ACT results can be affected by factors other than UFH, including hemodilution, hypothermia, anemia, thrombocytopenia, the presence of platelet inhibitors, severe hypofibrinogenemia, low antithrombin levels, and deficiency of other coagulation factors [113].

Advantages of ACT are seen in its low cost, point-of-care utilization, the small amount of blood needed, and its results being available within a few minutes. Finally, it presents the global in vitro functional test of the clotting system, incorporating the platelets and other molecules with a role in coagulation [21].

However, this method is losing its popularity as recent studies found a poor correlation between ACT, heparin doses, and anti-Xa activity, recommending instead the use of anti-Xa activity for anticoagulation monitoring [114].

### 5.2. Activated Partial Thromboplastin Time (aPTT)

The aPTT assesses the intrinsic and common pathway of coagulation and it is a well-known parameter for heparin therapy monitoring, except in cases when high dosing is required (e.g., cardiopulmonary bypass, ventricular assist device implantation, cardiac catheterization). For performing the aPTT test, citrated plasma is mixed with calcium and silica (ellagic acid) to initiate clot formation and the clot is detected by optical (change in blood density) or mechanical (the movement or oscillations of a steel ball in the test solution) methods [114]. The recommended therapeutic range for the prevention of venous thromboembolism is 1.5 to 2.5 times the patient’s baseline aPTT (40–80 s) [115]. However, this range was never validated in randomized clinical trials or in patients receiving ECMO support [116].

The use of aPTT monitoring is based on the assumption of a linear relationship between UFH dose and aPTT. However, aPTT may be influenced by the consumption of coagulation factors in the setting of thrombosis or bleeding, antithrombin levels, lupus inhibitor, and elevated C-reactive protein, factor VIII, or fibrinogen, often present in critically ill and ECMO patients. Moreover, the presence of a wide variability in the aPTT reagents sensitivity and individual laboratory methods is well described. This diversity may result in different aPTT results depending on the employed method, and each laboratory should set its own aPTT ranges for safe and reliable anticoagulation monitoring (limiting potential comparison of studies). An alternative method of UFH monitoring is anti-Xa activity, but whether this method is more appropriate than aPTT remains controversial [21,117].

The main advantages of aPTT monitoring are the wide availability, the dual use for UFH and DTI monitoring, and the availability of point-of-care tests using the whole blood [116,118].

### 5.3. Anti-Factor Xa Activity Levels (Anti-Xa)

Anti-Xa assays are gaining popularity and becoming an important component of UFH titration for ECMO support, with an increasing number of centers using this testing in addition to standard ACT or aPTT monitoring [114,119,120]. It measures the ability of heparin-bound antithrombin to inhibit factor Xa, providing information on the heparin effect rather than its concentration. The mechanism of action can be explained by the formation of an inactive antithrombin–Xa complex as a direct effect of heparin, with residual Xa left in the blood sample. This residual factor Xa is further measured, using either a chromogenic or clotting-based assay, and its levels are inversely proportional to the heparin concentration in the sample.

From the method used, the main limitations arise. Hyperlipidemia, hyperbilirubinemia, high plasma-free hemoglobin, or low antithrombin values can all falsely lower the measured Xa levels. Both elevated bilirubin and free hemoglobin (hemolysis) may be frequently seen during ECMO support, limiting its practical application. Moreover, as this is a plasma-based assay, it excludes the role of fibrinogen and platelet function in forming a stable clot. Given the above, false low antifactor Xa levels or underestimation of the role of platelets may lead to over-anticoagulation by increasing the heparin dose with the potential risk of adverse events [21,58,121]. Moreover, there is still room for improvement in the sense of its availability and cost.

Moreover, multiple studies showed that the anti-Xa assay correlates better with heparin concentration when compared to both ACT and the aPTT, especially in critically ill patients [114,119,120]. It may also mitigate some limitations of aPTT, for example, the presence of lupus anticoagulant or elevated C-reactive protein. A recent meta-analysis of 26 observational studies with 2086 patients compared the use of anti-Xa with the time-guided anticoagulation strategies (ACT, aPTT, clotting times from viscoelastic methods) by observing the mortality and incidence of bleeding and thrombotic events during ECMO support [122]. The authors concluded that anti-Xa-based anticoagulation monitoring was associated with decreased mortality, fewer bleeding events, and no increase in thrombotic events with respect to the study limitations (the nature of included studies and only a small fraction of adult patients) [122]. Moreover, Descamps et al. reported that the mean anti-Xa activity is an independent risk factor for bleeding complications in ECMO patients [120].

The typical therapeutic range for UFH is from 0.3 to 0.7 IU/mL [21]. Same as in the case of aPTT, this rather arbitrary range is still not validated in patients receiving ECMO support and is based on the therapy of non-ECMO patients [123].

The newly reported and promising feature for in vivo real-time monitoring of anti-Xa levels based on the microdialysis-coupled microfluidic system generated comparable results to those from the conventional assay. This method was tested in a small animal model and still needs further development until it can be tested in humans [124].

### 5.4. Viscoelastic Testing

Viscoelastic methods (i.e., thromboelastography system-TEG^®^, rotational thrombelastic system-ROTEM^®^, Sonoclot^®^, ClotPro^®^, etc.) provide a real time and holistic view of ex vivo coagulation. They include the evaluation of a clot initiation, strength, and stability (breakdown of the fibrin clot, fibrinolysis) covering the major coagulation components usually measured with separate coagulation tests. This point-of-care method provides the first results within five to ten minutes, saving valuable time in initial management [118]. The basic mechanism of function is the assessment of the physical clot characteristics during the progression of the whole blood sample from a liquid to a gel state. This is possible either by measurement of clot resonance frequency or clot shear modulus [125]. Results are finally presented in a live graphical trace, with different parameters corresponding to different hemostasis contributors [125].

Viscoelastic tests offer the broadest available in vitro coagulation testing, using whole blood samples and modern point-of-care methods. However, they still have limited availability, high costs, and limited data on correlation with conventional UFH monitoring and clinical outcomes. Moreover, first-generation models depend on manual pipetting of blood samples, which may be time consuming and have the potential for errors. Recently, ready-to-use cartridges are available, which are easy to use and save time [118].

The use of viscoelastic hemostatic assays is recommended for the guidance of coagulation factors and blood product substitution in patients with hemorrhagic diathesis, usually once a day in the case of ECMO patients [116]. Recent observations showed that the hypercoagulable state, as assessed by viscoelastic methods, may predict the risk of thrombotic adverse events. Although the evidence on the use of TEG^®^ and ROTEM^®^ in anticoagulation monitoring and ECMO patient management is increasing, it still exhibits differing results regarding the prediction of thrombosis and bleeding [116]. A recent prospective observational study reported a moderate correlation of INTEM CT (ROTEM^®^) with traditional tests, which is superior to TEG^®^. However, this study was limited by a small sample size (25 patients) and comparison to only aPTT and ACT, as standard coagulation monitoring [126]. Therefore, future clinical studies comparing viscoelastic methods with traditional monitoring are warranted.

### 5.5. Antithrombin Monitoring and Substitution

Antithrombin is a small glycoprotein and a natural serine protease inhibitor produced in the liver, with a half-life of 2 to 3 days. It can inhibit all procoagulant proteases of the clotting cascade, but it inhibits thrombin and factors Xa and IXa to the greatest extent [127]. Antithrombin has a low anticoagulant activity in its natural form and it can be enhanced more than 1000 times in presence of heparin [114].

The optimal antithrombin activity for patients undergoing ECMO support and receiving UFH is still unknown. However, if the activity of anti-Xa is not increasing despite the rise in the UFH dosing, the concentration of antithrombin should be checked and substitution may be considered [58]. The substitution threshold differs between centers (ranging from <30% to <80%, and most commonly when the level of antithrombin falls under 70%) and can be employed using commercially available antithrombin or frozen plasma, which results in a significant volume substitution (1 mL of plasma contains 1 unit of antithrombin) [58,128]. A randomized controlled trial of antithrombin supplementation during vv-ECMO support found that its replacement does not decrease UFH requirement, transfusion need, or the incidence of hemorrhage and thrombosis [129]. In conclusion, additional evidence is still needed before recommending routine antithrombin monitoring and substitution, as this may be associated with a significant increase in the cost of care with an unclear benefit for patients.

In summary, anticoagulation monitoring is still performed in vitro, and there is no available option for in vivo testing. In vitro testing limits coagulation monitoring by not considering other components of the complex patient coagulation system, like an endothelial response or even a blood–artificial-surface response in the case of ECMO support, but only tests the capability of ex vivo whole blood clotting. Moreover, all plasma tests miss the effect of other blood components, like platelets or clot strength. Finally, clear recommendations on anticoagulation monitoring based on strong evidence are still missing. Finally, the idea of continuous anticoagulation monitoring is very complex and still far from a reality.

Several new technologies for point-of-care coagulation testing are in the development phase, including fluorescent microscopy, electromechanical sensing, photoacoustic detection, microfluidics, and nano/microelectromechanical systems. The new technological trends should focus on the evolution of rapid, highly accurate, and cost-effective coagulation point-of-care assays, which are even more accessible and user-friendly [118].

## 6. Cost of Anticoagulation

The hospitalization of patients requiring ECMO support for postcardiotomy and cardiogenic shock has the greatest costs and lowest survival, compared to other indications (acute respiratory failure, bridge to heart or lung transplantation) [130]. The reported cost of ECMO support in the USA from a hospital-cost perspective was USD 318,187 [131]. The mean total costs of support in non-US studies ranged from USD 22,305 to USD 161,532.

However, the cost of anticoagulation is a smaller fraction of the total costs. In a study from the USA, the average cost of therapy with argatroban was USD 7091.98, and with UFH it was USD 15,323.49 (including costs of the drug, the substitution of blood or coagulation products, and laboratory tests for monitoring) [132]. The difference in cost is mainly attributed to the antithrombin substitution in the UFH group. Another study from Germany reported on the cost of anticoagulation, providing direct cost of drugs, HIT diagnostics, and blood products [90]. They found argatroban (EUR 26) to be more expensive per day on ECMO support than UFH (EUR 0.9). The costs of substituted blood products per ECMO day did not show a significant difference (argatroban EUR 28 and UFH EUR 34), and the total costs of anticoagulation per ECMO day, including HIT testing, tended to be higher in the argatroban group (EUR 63 vs. EUR 40) [90].

Coughlin et al. reported the daily cost of UFH (USD 10; USD 450 with once daily anti-Xa analysis), argatroban (USD 167.33), and bivalirudin (USD 734) [89]. They report further on the approximate cost of other components that may be employed during ECMO support, for example, (1) laboratory tests: aPTT: USD 60; TEG/ROTEM: USD 15; anti-Xa assay: USD 440; antithrombin level: USD 480; HIT diagnostic: USD 363; the serotonin release assay: USD 332; (2) medications: recombinant antithrombin with 3000 units: USD 7000; UFH with 1000 units: USD 0.27; argatroban: USD 3.32/mg (0.5 mcg/kg/min); bivalirudin: USD 3.37/mg (0.1 mg/kg/h) [89].

Finally, despite the higher DTI cost, its overall cost may be comparable with UFH if taken in the context of additional monitoring, required substitutions, and complications [89].

There is a paucity of evidence on the cost and cost/benefits of different extracorporeal circuits (coated and noncoated). The cost evaluation studies observed the improved outcome and consequent potential reduction in costs. However, the evidence regarding comparisons of different approaches is still limited and originates mostly from the cardiopulmonary bypass studies. Mangoush et al. reported that heparin-bonded circuits may reduce the re-sternotomy rate, the need for transfusion, the duration of ventilation, intensive care unit, and hospital stay, with a potential of cost saving [133]. One meta-analysis from 1998 compared the clinical outcomes and costs of heparin-bonded circuits and reported a cost saving of USD 3231 for covalently bonded circuits, mostly due to improved clinical outcomes such as reduced length of hospital stay and the need for transfusion [134]. Therefore, heparin-bonded circuits have the potential to improve resource utilization in patients undergoing cardiac surgery. The sparsity of evidence on the cost effectiveness of different ECMO circuit-coating approaches should be addressed in future studies.

## 7. Future Perspectives and Conclusions

Despite immense research and development in the field of ECMO circuit technology in the last decades, a need for systemic anticoagulation therapy still exists. The potential improvement of the clinical outcomes of patients undergoing ECMO support depends on the underlying disease and comorbidities, the technology employed, and on anticoagulation [16].

Heparin-coated circuits are used as the state-of-the-art in ECMO support, and many ECMO centers employ them as a standard [135,136]. However, the surface coating does not eradicate the risk of thrombosis or bleeding due to the potential of systemic anticoagulation reduction [137].Extensive research is directed toward the development of new artificial surfaces in two main directions: the use of synthetic and natural polymers for the surfaces coating or their endothelialization. However, none of the non-heparin coatings showed superiority to heparin coatings, making heparin coatings the most popular in everyday practice [138].

Different surface coatings, such as heparin, albumin, phosphorylcholine, polyethylene glycol, and poly-2-methoxyethylacrylate, have been developed to minimize the potential of thrombus formation during ECMO support (Table 2) [138]. Moreover, research on improving the hemocompatibility of commercially available membranes showed that grafting of the polymer brushes with the technique of single electron-transfer living radical polymerization can reduce recalcification time, reducing the adhesion of leukocytes and platelets [137]. However, the tests were performed on the same polymer but in different circumstances, material thickness, and configuration, which may not be useful in clinical practice. Cornelissen et al. have proved that coating of oxygenator membranes with fibronectin enhances endothelial cell attachment [139]. Covering the membrane artificial surfaces with titanium dioxide may aid bonding of endothelial cells, allowing the development of mono-layered endothelium [140]. Finally, a new enduring and biocompatible ECMO pump system was successfully tested in animals with no clot formation within the centrifugal pump [141]. The mentioned studies should be interpreted with caution, as they are limited by in vitro testing. The artificial circumstances in which these experiments were conducted cannot be compared to the real-life clinical application. Further research and development of novel surfaces, including their adaptation for human use is warranted.

Published studies are reporting the importance of developing the ultimate biomembrane, which will mimic healthy vascular endothelial tissue. Nitric oxide (NO) and prostacyclin are examples of substances that, once integrated into the artificial surface, can modify the circuit, making it more similar to the endothelial tissue. Nitric oxide is a strong inhibitor of platelet activation and adhesion, influencing the fluidity of blood. Earlier research in animal models showed that NO coating may prevent platelet consumption and thrombus formation while preserving platelet function [142,143]. Moreover, there are reports on NO-releasing coatings for use in vascular stents and grafts or extracorporeal circuit tubing, but evidence for oxygenator membranes is still lacking. El-Ferzli et al. developed a NO-releasing peptide amphiphile nanomatrix, which showed a significant reduction in platelet adhesion on a small hollow fiber oxygenator membrane, without affecting gas exchange [144]. However, the experimental circumstances under which the testing is conducted may be far away from clinical application, still limiting its use in humans. The addition of NO to artificial membranes may also decrease the complications related to platelet dysfunction, the incidence of postoperative bleeding, and the need for blood product transfusion [145,146]. Finally, ongoing research is focusing on other types of membrane and circuit coatings and includes C1-esterase inhibitors, tethered liquid perfluorocarbon, and zwitterionic coatings [137,147,148]. More detailed information on the novel surfaces in ECMO circuits can be found in the review from Ontaneda and Annich, or the comprehensive literature on the present and future perspectives of surface coatings [57,138,149]. An overview of the oxygenator and tubing surface modifications in extracorporeal membrane oxygenation is presented in Table 2.

**Table 2:** Overview of the oxygenator and tubing surface modifications in extracorporeal membrane oxygenation.

Surface Modification	Representative (Manufacturer)	Mechanism of Action and Main Characteristics
**Biopassive coatings**		
Albumin and recombinant human albumin	X-EED (Xenios), Safeline (Maquet)	Passivation as the main mechanism of action. One of the first proteins used for coating; increases the hydrophilicity; reduces platelets and fibrinogen concentration on the surface; has potential to reduce complement activation
Poly-2-methoxyethylacrylate (PMEA)	X-coating (Terumo)	Reduced platelet adhesion and protein denaturation as the main mechanism of action. Inferior hemocompatibility compared to Bioline, Phisio, and Trillium; causes transient leukopenia; reduction in platelet and leukocyte activation and adhesion, reduced coagulation and complement activation, reduced inflammation markers. Compared to other coatings, observed increase in ventilator time and chest tube output
Polyethylene glycol	E8 (Nipro)	Hydrophilicity as the main mechanism of action. Reduction in aggregation and reduction in inflammatory response
Phosphorylcholine	AGILE (Eurosets), Phisio (Sorin)	Cell membrane mimic as the main mechanism of action. Nonthrombogenic; reduced platelet and fibrinogen binding through GPIIb receptor; reduced complement activation; antifouling properties; reduction in other inflammatory markers; may increase immune cell response (T-cells)
*Under development*		
Tethered liquid perfluorocarbon (omniphobic surfaces)	Tethered liquid perfluorocarbon	Slippery liquid barrier layer is the main mechanism of action. Low adsorption and adhesion of plasma proteins; reduced physico-chemical interactions with the surface; in vitro experiments showed improved prevention of thrombus deposition compared to standard heparin-coated surfaces; data origin from extracorporeal circuits and animal studies
Zwitterionic polymers	SB-co-methacrylic acid block copolymer, 2-methacryloyloxyethyl phosphorylcholine,	Hydrophilicity as the main mechanism of action, originally inspired by phosphorylcholine. Decreased bovine serum albumin and fibrinogen absorption and platelet adhesion; prolonged aPTT compared to pristine surfaces under static conditions: data origin from extracorporeal circuits and animal studies
**Bioactive coatings**		
Heparin	Cortiva BioActive Surface (Medtronic), Rheoparin (Xenios), Hepaface (Terumo)	Heparin as anticoagulant. Reduced thrombin production; platelet binding; and D-dimer production; reduced inflammatory response and complement activation; majority of evidence originate from the cardiopulmonary bypass; danger of HIT II; heparin leach; oxygenator swelling and occlusion in case of ionic binding (covalent binding without leaching of heparin)
*Under development*		
Heparin-based coatings	T-NCVC coating	Hydrophobic properties; limited heparin leaching; high antithrombogenicity and long-term durability; data origin from extracorporeal circuits and animal studies
	Heparin coupled polyethylene glycol grafted polysulfone membranes	Improvement in hemocompatibility (albumin and fibrinogen adsorption and platelet adhesion) compared to noncoated membranes; no studies with comparison to heparin-only coated membranes available
	Antithrombin-heparin covalent complex	Higher antithrombotic activity; inhibition of clot-bound thrombin and longer half-life in the circulation compared to heparin
Nitric oxide releasing coatings Combination of nitric oxide and other anticoagulants (argatroban)		Inhibition of platelet and leucocyte activation; inhibition of platelet adhesion; improved endothelial mimetic microenvironment; lower fibrinogen consumption. Improved hemocompatibility in combination with other anticoagulants; data origin from extracorporeal circuits and animal studies; molecule leaching with nitrosamines release in the blood; the nitric oxide storage last for only 4 weeks; no commercial use until now; undergoing studies on endogenous nitric oxide reservoirs (e.g., nanotechnology, metal-organic frameworks, etc.)
Complement inhibitors	C1- esterase inhibitor coating	Improved reduction in factor XIIa activity compared to heparin coatings; C1- esterase inhibitor/heparin coating showed promising results in platelet adhesion and fibrin networks inhibition; data origin from extracorporeal circuits studies
**Combination**		
Heparin and albumin	Bioline (Maquet)	Anticoagulation and passivation as the main mechanism of action. Improved hemocompatibility; reduced complement activation and reduction in other inflammatory markers
Polyethylene oxide/sulphate/sulfonate groups with or without heparin	Balance and Trillium Biosurface (Medtronic)	Hydrophilicity, negative charge, and anticoagulant mechanism of action. Reduced protein (both fibrinogen and albumin) and bacterial adhesion; reduction in inflammatory markers; reduction in bleeding events; mimicking endothelium; negative charge dependent platelets repletion and inhibition of thrombin; preserved platelet count; increases the stroke rate in cardiopulmonary bypass; evidence from small and single center studies, missing long-term evaluation

Adapted from: [57,80,138,142,149,150,151].

Regarding the novel anticoagulant therapy, all currently available drugs have limitations and there is ongoing research aimed at finding the “ideal” anticoagulant. The ideal anticoagulant should have predictable pharmacokinetics and anticoagulant effects, a wide therapeutic range (removing the need for monitoring and reducing the risk of adverse events), a rapid onset/offset of action, an available antidote, parenteral and oral pharmacological formulation, and an affordable price [93]. Despite this, we still require robust data on the true extent of the anticoagulation needed; in keeping with the motto “as much as needed and as little as feasible”.

Emerging preclinical data focusing on the role of antibodies targeting factors XI and XII showed improved efficacy and safety in animal models, but data in humans are still missing [53,152,153]. Factor XII seems to be an ideal target since its deficiency is not associated with abnormal hemostasis and protects from thrombosis [153]. Recently, fully human factor XIIa neutralizing antibodies (3F7 and 5C12) were isolated with encouraging results [52,53]. The neutralizing antibodies had similar anticoagulant activity but with a drastically better effect on hemostasis. In addition, another selective factor XIIa inhibitor coded as FXII900 showed efficient anticoagulation without increased risk of bleeding in the rabbits´ ECMO setting [154]. Moreover, blocking factor XIIa could lead to the reduction in the C1 component of complement and lesser activation of the kallikrein/kinin system, preventing the generation of bradykinin and reducing inflammation [153]. Lastly, the humanized antifactor XI antibody (AB023), which blocks factor XIIa-mediated activation of factor XI, showed promising results in phase 1 human trials, on healthy volunteers [155]. Thus, we need to await more results from future phase 2 trials. Moreover, clinical studies should further narrow down the reported range of thrombosis incidence, which is perceived as one of the most important indications for anticoagulation. This holds especially true as bleeding increases mortality, but thrombosis does not [16].

Despite previously published anticoagulation guidelines, there is still not enough evidence for strong recommendations on a standardized method to monitor and conduct anticoagulation in ECMO patients [58]. The recommended and most-used monitoring methods have serious limitations for the evaluation of anticoagulation, especially in critically ill ECMO patients. However, until more adequate methods are available, standardized monitoring should be supported with a combination of tests like anti-Xa assay, viscoelastic methods, or platelet function tests. From our perspective, a leap in the development of anticoagulation could be in vivo and real-time monitoring of hemostatic capacities, with the first steps towards conceptualization already having been taken [124].

Given the above, developments in anticoagulation and ECMO research bring us closer to the idea of a perfect anticoagulant and monitoring modality, including ECMO circuits, and extensive research is warranted to translate the effects proved in animal studies into clinical applications. Whilst it is difficult to isolate the relative contribution of the particular patient- and drug-related factors for complications during ECMO support, failure to do so may result in a missed opportunity for intervention.

We believe that the best approach to anticoagulation is patient-individualized anticoagulation. The optimal anticoagulation titration, monitoring, and supplementation of blood products or factors should be conceptualized according to the overall inflammatory/disease and hemostatic state of the patient, as supported by combined laboratory and clinical evaluations. Anticoagulation therapy should be employed based on the most recent recommendations, including the assessment of the individual patient´s risk of adverse events.

## Data Availability

Not applicable.
